# An Overview of Diabetic Foot Ulcers and Associated Problems with Special Emphasis on Treatments with Antimicrobials

**DOI:** 10.3390/life12071054

**Published:** 2022-07-14

**Authors:** Mirza Shahed Baig, Ahmadi Banu, Mehrukh Zehravi, Ritesh Rana, Sushil S. Burle, Sharuk L. Khan, Fahadul Islam, Falak A. Siddiqui, Ehab El Sayed Massoud, Md. Habibur Rahman, Simona Cavalu

**Affiliations:** 1 Department of Pharmaceutical Chemistry, Y. B. Chavan College of Pharmacy, Aurangabad 431001, India; mirzashahed2@yahoo.co.in; 2 Department of Pharmacology, Vishnu Institute of Pharmaceutical Education & Research, Narsapur 502313, India; ahmadibanu@yahoo.co.in; 3 Department of Clinical Pharmacy Girls Section, Prince Sattam Bin Abdul Aziz University, Alkharj 11942, Saudi Arabia; mahrukh.zehravi@hotmail.com; 4 Department of Pharmaceutics, Adarsh Vijendra Institute of Pharmaceutical Sciences, Shobhit University, Gangoh, Saharanpur 247341, India; rvritesh1719@gmail.com; 5 Department of Pharmacology, Smt. Kishoritai Bhoyar College of Pharmacy, Kamptee, Nagpur 441002, India; drsushilburle@gmail.com; 6 Department of Pharmaceutical Chemistry, MUP’s College of Pharmacy (B Pharm), Degaon, Risod, Washim 444504, India; falakarjumand26@gmail.com; 7 Department of Pharmacy, Faculty of Allied Health Sciences, Daffodil International University, Dhaka 1207, Bangladesh; fahadulislamdiu@gmail.com; 8 Biology Department, Faculty of Science and Arts in Dahran Aljnoub, King Khalid University, Abha 62529, Saudi Arabia; ehabma@kku.edu.sa; 9 Research Center for Advanced Materials Science (RCAMS), King Khalid University, Abha 61413, Saudi Arabia; 10 Agriculture Research Centre, Soil, Water and Environment Research Institute, Giza 3725004, Egypt; 11 Department of Global Medical Science, Wonju College of Medicine, Yonsei University, Wonju 26426, Korea; 12 Faculty of Medicine and Pharmacy, University of Oradea, Pta 1 Decembrie 10, 410087 Oradea, Romania

**Keywords:** diabetic foot ulcers, diabetes mellitus, Wagner grade, diabetic neuropathy, antimicrobials, biofilms

## Abstract

One of the most significant challenges of diabetes health care is diabetic foot ulcers (DFU). DFUs are more challenging to cure, and this is particularly true for people who already have a compromised immune system. Pathogenic bacteria and fungi are becoming more resistant to antibiotics, so they may be unable to fight microbial infections at the wound site with the antibiotics we have now. This article discusses the dressings, topical antibacterial treatment, medications and debridement techniques used for DFU and provides a deep discussion of DFU and its associated problems. English-language publications on DFU were gathered from many different databases, such as Scopus, Web of Science, Science Direct, Springer Nature, and Google Scholar. For the treatment of DFU, a multidisciplinary approach involving the use of diagnostic equipment, skills, and experience is required. Preventing amputations starts with patient education and the implementation of new categorization systems. The microbiota involved in DFU can be better understood using novel diagnostic techniques, such as the 16S-ribosomal DNA sequence in bacteria. This could be achieved by using new biological and molecular treatments that have been shown to help prevent infections, to control local inflammation, and to improve the healing process.

## 1. Introduction

More than 415 million people throughout the world are diagnosed with diabetes, and that number is expected to climb to 640 million (1 in 10) by the year 2040, according to the International Diabetes Federation’s 2015 study (IDF 2015). A further 12 percent of global health budgets are allocated to the treatment of people with diabetes (USD 673 billion) [1,2]. People with diabetes are more likely to suffer from skin wounds, particularly chronic ulcers, due to neuropathy (nerve damage) and arterial (blood vessel) disease or trauma. Peripheral neuropathy (nerve dysfunction in the feet) and peripheral artery disease (both) are common in persons with diabetes. People with diabetes have immune system impairments that have yet to be discovered, limiting their ability to avoid or treat illnesses. Foot ulcers are a common complication in people with diabetes because they are more likely to develop in persons with the disease [3]. It is estimated that a person with diabetes’ lifetime risk of developing a foot ulcer is 25%, with an uninfected ulcer costing EUR 10,000 and an untreated ischemic ulcer costing EUR 17,000 in 2008 [4]. When these wounds become clinically infected, they cause a large amount of morbidity. A person with diabetes is amputated of a lower limb every 20 s, on average, according to worldwide statistics. When at least two typical signs or symptoms of inflammation (pain or tenderness, warmth, redness, and swelling) or purulent discharges appear in a diabetic foot ulcer, an infection has occurred (pus) [5]. Patients with diabetes now spend more time in the hospital due to foot issues than any other diabetic complication. In patients with diabetes, diabetic foot infections, particularly those that extend to the bone, are the primary cause of lower-extremity amputation, which results in an increased risk of mortality and a higher cost burden [6]. To avoid these bad outcomes, it is essential to prevent foot infections or, if that is not possible, to take care of wounds that have not been treated. There are a lot of methods to provide antimicrobial therapy: intravenous injections, injections into muscles, and other means. One of the most popular kinds of antibiotic treatment is to administer the drugs topically, in other words, locally. Even if the patient has neuropathy or vascular diseases, it is frequently difficult to tell whether a diabetic foot ulcer is infected. Furthermore, even in clinically uninfected wounds, the sheer presence of microorganisms might delay wound healing, especially if they are pathogenic or present in huge numbers [7]. Some doctors believe that antibiotics (especially topical ones) may effectively treat high-risk wounds that are clinically uninfected [8,9].

DFU treatments should follow a multidisciplinary approach that uses various diagnostic tools, is performed by various specialists, and requires years of experience in treating the condition. Patients must be educated to prevent amputations, and new categories must be used to guide treatment [10,11]. To learn more about DFU microbiota, it will be required to apply cutting-edge diagnostic tools such as the 16S ribosomal DNA sequence in bacteria. In addition to wound characteristics, local epidemiology-based antibiograms, personalized treatment, regular debridement, periodic wound assessment, and dressing changes, DFU is said to have a range of distinctive properties [12]. Infection prevention, local inflammation management, and cicatrizing efficiency may all be improved by bio-molecular therapy and many other characteristics of the human body. In particular, this survey will look at the most recent developments in antimicrobial treatments, such as dressings; topical therapies; medications; debridement techniques; cellular, gene, and molecular therapies; plant extracts; antimicrobial peptides; growth factors; devices; and energy-based treatments.

## 2. Methodology

**Figure 1:**
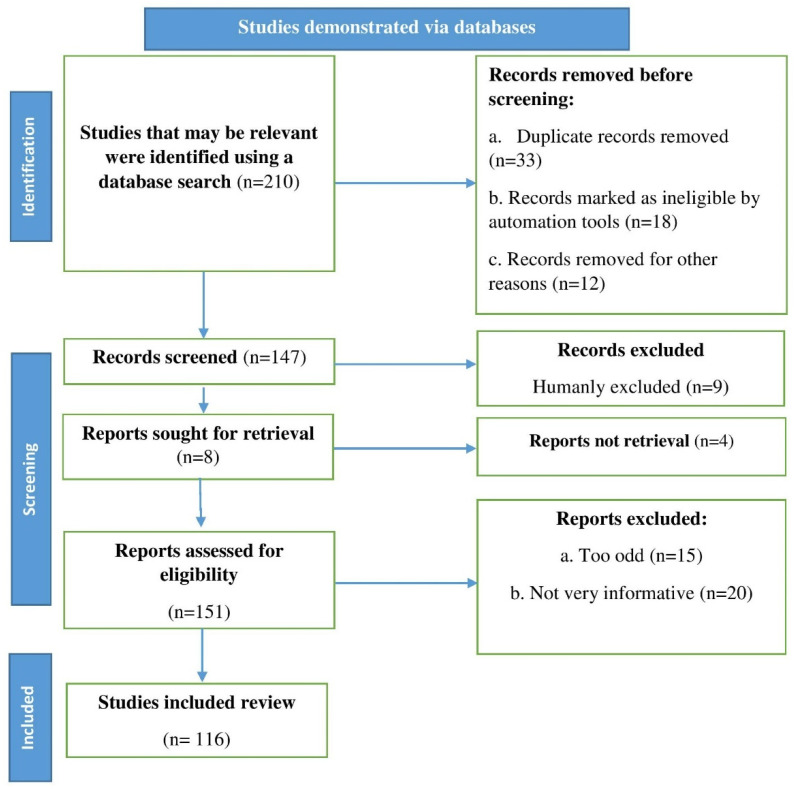
A flowchart illustrating the steps required for choosing published data to be used in the current study is shown; n = number of literature reports.

The following databases were used: PubMed, Scopus, and Web of Science. The terminology diabetic foot ulcers, antimicrobials, biofilms, and multidrug-resistant were used. Up until 2022, English research reports, reviews, and original research articles were chosen and examined. According to Page et al.’s [13] guidelines, an algorithm that followed the flowchart in Figure 1 and included all of the processes and requirements for selecting the necessary literature was utilized.

## 3. Diabetic Foot Ulcers (DFUs)

DFUs, which are usually skin ulcers that progress across the entire lower limb with various degrees of peripheral vasculopathy and neuropathy, morbidity, disease, death, and psychosocial distress. Osteomyelitis and gangrene also accompany DFU. With extreme DFU, amputation of a significant leg is often used to manage long-term recurrence [14,15]. That is why there are so many various categorization systems for when a foot ulcer responds to therapy. As of yet, it has not been proven to be a commercial success. Therapeutic data-recording devices are mostly a matter of convenience, rather than clinical or theoretical usefulness, for most people with diabetes [16]. In order to determine the severity of an ulcer, the presence of osteomyelitis or gangrene, and the need for an amputation, the Wagner ulcer classification system uses the following criteria: Wagner grade 0: intact skin; Wagner grade I: superficial ulcer of skin or subcutaneous tissue; Wagner grade II: ulcers extend into tendon, bone, or capsule; Wagner grade III: deep ulcer with osteomyelitis or abscess; Wagner grade IV: partial foot gangrene; and Wagner grade V: whole foot gangrene [17].

An amputation is now required in 90 percent of patients with diabetic foot ulcers with Wagner grade III or above. Approximately 45 percent of patients with diabetic foot ulcers in China have a Wagner grade of III or above, with amputation rates from 18 to 28 percent, according to a nationwide study. Patients with DFU had mortality rates of 11% or higher. The 5-year mortality rate of DFU in Tianjin, China, was found to be 32.7 percent. In the United States, the cost of treating DFU in 2017 was USD 727 billion, while in China, it was USD 110 billion [17,18]. Including the fact that endovascular operations and vascular bypass surgery are the recommended treatments for ischemia foot ulcers, 40% of patients with DFU and serious limb ischemia may not follow the criteria. Consequently, amputation is often considered the safest choice for many patients with DFU. In the five years after amputation, the death rate was around 25–50 percent. Traditional therapy has recurrence rates of 40 percent after one year, 60 percent after three years, and 65 percent after five years. Because of this, new treatments are urgently needed to improve DFU healing and limb preservation rates [19].

**Figure 2:**
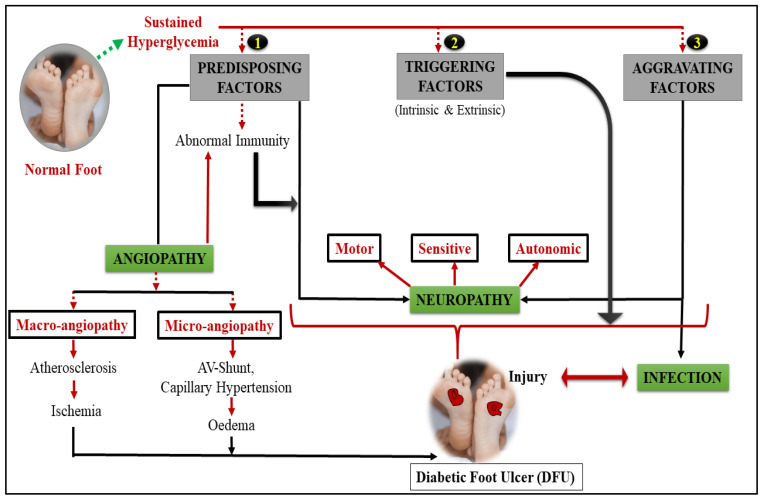
DFUs are caused by a combination of risk and predisposing factors.

All patients with osteomyelitis must have their DFUs discarded. If a bone sample is indicated in the case of a suspected fracture, C-reactive protein (CRP), ankle–brachial index (ABI), and X-ray/MRI imaging should all be performed. Primary care settings are constrained in their ability to conduct regular health evaluations due to the lack of time available for foot inspections. Neuropathy; peripheral artery disease (PAD); immune system variables; and in certain instances, recurring external or mild damage are among the risk factors for diabetes (which lead to skin breakdown and ultimately to the development of infection). Toe deformities (such bunions and hammertoes) are also considered risk factors since they may produce trigger points on the foot (potential locations for ulceration). Figure 2 illustrates the many risk and predisposing variables that might lead to DFUs. Patients with neuropathy are thought to have more mechanical pain than people with diabetes without the disease. Inflammation is the most common cause of amputation, which occurs in people with severe diseases, further tissue loss, and organ failure across the body. Patients with anemia (a hemoglobin level below 11 μg/dL), those who are older, and those who suffer from PAD are at greater risk of infection and, as a result, of amputations [20,21].

## 4. Pathophysiology of DFUs

Diabetic neuropathy and PAD are the major causes of DFUs, with trauma acting as a starting trigger. At various points in the healing process, both of these factors contribute to the development of ulcers.

### 4.1. Diabetic Neuropathy

Neuropathy in the sensitive, motor, and autonomous nerves is caused by oxidative stress in the nerve cells caused by hyperglycemia. When the hexosamine metabolic route is activated, it reduces the amount of aldose reductase and sorbitol dehydrogenase produced by the polyol metabolic pathway, which absorbs nicotinamide adenine dinucleotide phosphate (NADPH). These enzymes are responsible for the transformation of glucose into sorbitol and fructose [22]. Myoinositol synthesis in nerve cells reduces as these sugar products pile up, resulting in decreased neuronal conduction, increased levels of antioxidants such glutathione, and an increase in reactive oxygen species (ROS) formation [23]. In addition to the increased flow of hexosamine and polyol pathway, the altered development of substance P, nerve growth factor, and calcitonin gene-related peptide all lead to additional nerve damage and ischemia [24]. For example, when there is damage to motor neurons in the foot muscles, an imbalance in flexor and extender muscles might occur, resulting in anatomical deformity and skin ulcers. Skin breakdown may occur as a consequence of damage to the autonomic nervous system because of a decrease in sweat gland activity and an inability to moisturize the feet [25]. If peripheral sensation in the skin is reduced, it is possible that patients will be more cautious about acquiring foot wounds because the skin is less likely to contain intra-epidermal nerve fiber endings of the afferent A-delta and C-fibers, the majority of which are nociceptor nerve endings that are only stimulated by pain. Diabetes-related neuropathic illnesses, such as vitamin B12 insufficiency, alcohol toxicity, and renal failure towards the end of life, might exacerbate this condition. Epidemiological studies suggest that fat lipoproteins, high blood pressure, and smoking all have roles in the development of PAD. Charcot’s foot, the most well-known sign of motor neuropathy, is only one of several. It is crucial to keep in mind that the foot’s skin sheaths, tendons, and soft tissues make it vulnerable to infection (such as plantar aponeurosis and fascia) [26,27,28].

### 4.2. DFUs Pathogenesis: Immunological Involvement

The immune system of individuals with diabetes is characterized by a reduced healing response in DFUs. There are many examples, including T-lymphocyte apoptosis; proinflammatory cytokines; degradation of polymorphonuclear cell functions such as chemotaxis, adhesion, and intracellular killing; inhibition of fibrocyte proliferation; and impaired basal layer of keratinocytes with reduced migration of epidermal cells [27,28]. Bacteria, particularly aerobic Gram-positive *cocci*, such as *Staphylococcus aureus* (*S. aureus*) and hemolytic *streptococci*, flourish at high blood glucose levels. Carbohydrates, fibroblasts, and collagen synthesis are all affected by diabetes’ metabolic insufficiency as well as other structural inadequacies. Serum glucose concentrations more than or equal to 150 mL/dL were also considered indicative of immune system dysfunction. These traits are likely to lead to a long-term inflammatory disease [22,23].

### 4.3. PAD

Almost 80% of individuals with DFU already suffer from PAD [29]. When blood sugar levels are too high, they cause changes in the foot’s peripheral arteries, which begin at the cell level. The malfunction of endothelial cells is the most important aspect of microcirculation dysfunction. This is because endothelial cell dysfunction causes a decrease in the generation of vasodilators, most notably nitric oxide. Persistent vasoconstriction and hypercoagulation increase plasma thromboxane A2 levels, increasing the risk of ischemia and ulceration [30]. It is possible that the endothelium will show signs of reduced local angiogenesis, endocrine cell proliferation, basement membrane thickness, blood viscosity, changes in microvascular sound, and antioxidant potential. It might also show signs of reduced smooth muscle cell proliferation [31].

## 5. Infection of DFUs

“Infection” is defined as the invasion and proliferation of dangerous bacteria inside tissues, according to the international working group on the diabetic foot. Patients with diabetic foot infections (DFIs) are at greater risk of having a leg amputated and of experiencing a higher risk of death [32]. Ulcer complications such as DFU infections are common and serious. It is estimated that DFU infection is the cause of 80% of non-traumatic lower-limb amputations, with 50% of DFUs being compromised at the time of diagnosis. Some patients with DFI are hospitalized and given many doses of antibiotics. Skin infections may delay recovery and lead to systemic health issues if cared for incorrectly. Wound microbiology is a major factor in the onset of foot infection [33,34,35]. An organism’s level of microbiota as well as its ability to interact with other microorganisms are important considerations. When the number of bacteria per gram of tissue surpasses 105, the condition is referred to as an infection [36]. It is possible for skin commensal bacteria to colonize the wound left behind by DFUs, even when the wound is not infected since the host’s immune system has not yet been activated [36]. Triggers might be physical, chemical, or mechanical in nature. The DFU is sensitive to infection because of ischemia, neuropathy, edema, inflammation, and a reduced immune system [37]. It is possible to determine whether an ulcer has been infected by using recommendations issued by the Infectious Diseases Society of America (IDSA). The infection is detected if at least two of the following symptoms are present during a clinical examination: inflammation, induration, perilesional erythema, hyperesthesia, pain, local fire, and purulent exudate [38]. According to research, 78% of those who undergo DFU already suffer from PAD. Endothelial cell dysfunction is the most critical feature of microcirculation dysfunction, since it results in decreased production of vasodilators, particularly nitric oxide. Chronic vasoconstriction is caused by high plasma thromboxane A2 levels, which increases the risk of ischemia and ulceration [39,40,41].

### 5.1. Bacterial Species of the DFUs

**Figure 3:**
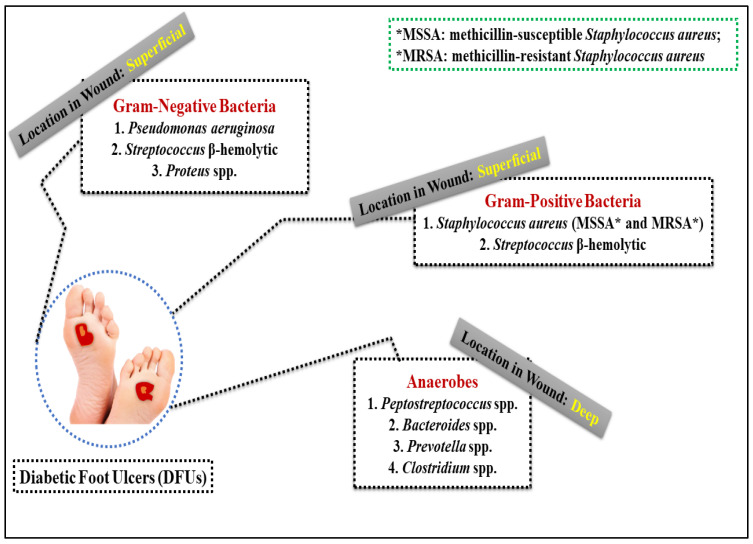
The most common bacteria detected in the DFUs.

The DFUs’ microbiome has been studied extensively. The organism’s immune system and physio-pathological features heavily influence the composition of this microbiota. Using molecular tools, researchers have found the polymicrobial nature of chronic wounds such as DFUs, which comprise Gram-negative and Gram-positive bacteria, as well as anaerobic bacteria and certain fungi [37]. In the past, conventional bacterial culture methods focused on a single bacterium, which was the only one present (Gram-positive bacteria). The microbiome of diabetes and non-diabetic ulcers differed, with Gram-negative and Gram-positive bacteria being found in different proportions. In a microbiological examination of DFI by another author, Gram-negative bacteria were found to outweigh Gram-positive ones (59 percent vs. 41 percent) [37]. Microorganisms in DFUs have “preferred locations”, which are defined by the amount of oxygen they take up when present. In contrast to anaerobes, which live deeper inside the niches given by aerobic oxygen intake, aerobic bacteria may be found at the surface, where oxygen levels are quite high [42]. While *Pseudomonas species* are the most commonly isolated Gram-negative bacteria, *Escherichia coli*, *Proteus species*, *Enterobacter species*, and *Citrobacter species* are the most commonly isolated Gram-positive bacteria. *S. aureus* was found in 72% of culture-positive samples in a microbiome analysis of fresh and chronic DFUs using 16S amplicon sequencing. Geography has a significant role in the genesis of DFUs [43]. Gram-positive aerobic *cocci* are the most common microbe in Western countries, whereas Gram-negative *bacilli* are more common in warmer climates (particularly Asia and Africa). Normal procedures yielded the most common bacteria in Mexico: *Staphylococcus aureus*. In Bangladesh, the most common bacteria in DFUs samples were *Pseudomonas* spp. (22/29 percent), *Enterobacter* spp. (22/7 percent), and *Staphylococcus* spp. (13/13 percent) [44]. India also had the highest percentage of Gram-negative infections (58.5%), indicating the prevalence of Gram-negative bacteria in Eastern countries [43]. Up to 95% of all instances of anaerobes found in severe diabetic wounds were caused by *Peptostreptococcus* spp., *Bacteroides* spp., and *Prevotella* spp. [45]. Figure 3 depicts the location of DFUs in relation to the most common bacteria detected in the wound. As a result, DFIs are more prone to develop larger, more frequent ulcers accompanied with ischemia, necrosis, or unpleasant odors [45]. 

### 5.2. Existence of Biofilms and Its Production in DFUs

The term “biofilm” refers to an assemblage of bacterial populations that is well organized and encased in a polysaccharide matrix. Chronic diabetic foot sores are made worse by the formation of biofilms [46]. Wound healing is slow and infection resistance is difficult to overcome because biofilm prevents the host’s immune system from accessing antimicrobial medications. *S. aureus* accounted for the bulk of biofilms, and bacteria that caused chronic DFUs were typically multidrug-resistant, according to a study [47].

Biofilms do not cause foot ulcers; rather, they are precipitating factors such as of peripheral neuropathy (the loss of defensive sensitivity), altered foot architecture, trauma, and Patch [48]. This causes the skin’s protective layer to break down in both cases. When pathogenic biofilms have developed in DFUs, they may be a cause of recurrent and reoccurring infections, prolonging the healing of the ulcer [48]. In vitro and animal studies have revealed that biofilms impede wound healing. While biofilms have been linked to delayed ulcer healing and chronic infections in the foot of a patient with diabetes, translational evidence from human therapeutic trials is lacking, prompting more research [49]. DFU biofilms have been studied extensively using DNA sequencing technology, which has provided a more complete view of the microbiota of diabetic feet [50]. The most common bacteria detected in DFUs with biofilm forms have been described in the diabetic foot literature, notwithstanding this fact. The majority of DFUs include polymicrobial biofilms. A large number of *staphylococci* and *streptococci* are found in the environment [51,52]. Fastidious anaerobics (particularly, those belonging to the Clostridiales Genus XI), *Corynebacterium* spp., and Gram-negative rods are among the bacteria usually found in the same foot ulcers (namely, *Klebsiella* spp., *Acinetobacter* spp., *Enterobacter* spp., *P. aeruginosa*, and *Escherichia coli*) [53,54].

## 6. Multidrug-Resistant Bacteria in DFUs

In diabetic foot ulcer research, drug-resistant species are overrepresented. *Methicillin-resistant Staphylococcus aureus* (MRSA) was prevalent among patients visiting a multidisciplinary Melbourne secondary treatment center in Australia, as was the case with various other populations. Among 653 specimens from 379 patients, MRSA was found in just 23% of cases [55].

In a French study in 2008, 188 individuals brought to the hospital with an untreated foot ulcer had their recovery rates monitored by the MDR [56]. Two-thirds of the ulcers were categorized from moderate to severe in the study, revealing their intricacy. It has been established that 70% of the ulcers are neuro-ischemic ulcers, with a fifth of the lesions being resistant to antibiotics [57]. Lower-limb amputation was more common in patients with MDR microorganisms than in those with non-MDR infections (35.6 percent compared to 11.2 percent). The majority of these amputations (87.5 percent) were moderate. Multivariate analysis, however, showed that the presence of MDR bacteria had minimal impact on healing time after adjusting for other factors [58,59,60].

## 7. Therapeutic Methods Used to Manage DFU Infections

DFIs may end in amputation of a section or more of a patient’s foot or leg, as well as in death in severe situations. An infected diabetic foot, particularly paired with ischemia, remains one of the most severe challenges in the management of DFUs. Due to the presence of bacteria, local and systemic cytokines are generated, which may lead to systemic inflammatory response and shock, underscoring the requirement of infection care for DFUs. Based on the intensity of the illness, a number of antimicrobial drugs and physical techniques are commonly utilized, ranging from topical and oral remedies for light and moderate infections to intravenous therapy for more severe infections. If an antibiotic treatment is started, it must be completed until all clinical symptoms have disappeared and test results have returned to normal. The wound should be frequently evaluated (at a dressing change or on a bi-weekly schedule) throughout infection management to assess the effectiveness of the treatment [61,62,63].

### 7.1. Removal of the Bacterial Biofilm (Debridement)

Because debridement eliminates both the bacteria biofilm and dead tissue from the lesion, it is essential in treating a foot ulcer infection. Tissue for microbiological culture and wound healing may be obtained from the wound, but it also allows for a more detailed evaluation of the wound [64]. The necrotic tissue that accumulates around a wound during the normal healing process is called necrotic debris. Debridement speeds up wound healing by removing dead tissue that would otherwise obstruct the growth of new tissue. Isotonic saline solutions must be used for wound cleansing and debridement prior to antibiotic treatment (0.9 percent NaCl) [65]. Sharp debridement typically reduces the bioburden of hyperkeratotic margins of plantar neurotrophic ulcers. Every seven to fourteen days, this procedure should be performed [66]. Active and autolytic debridement methods are used in the clinic. Surgical debridement, which removes dead tissue using a scalpel and tweezers while causing the wound bed to bleed, is an example of active debridement [67]. Hydro-surgical debridement involves the use of a solid stream of water to remove dead tissue. In outpatient settings, the use of ultrasound-assisted debridement is beneficial. In this procedure, low-frequency waves (25 kHz) and irrigation fluids are employed. As the moisture in the wound increases, natural tissue shedding occurs. This is commonly achieved by the use of hydrocolloids and hydrogels [68]. According to a review, there was no difference in wound duration between clostridial collagenase ointment (CCO) and traditional hydrogel treatment at six and twelve weeks [69,70].

### 7.2. Dressings

Wound dressings are an essential aspect of treatment for DFUs right now. To better treat DFUs, clinicians have come to appreciate the need for the use of wound dressings that promote faster healing, prevent the spread of bacteria, and enhance the overall healing process [71]. Silver dressing has been shown in several trials to be helpful in treating DFUs. Researchers have shown that silver ion dressings may destroy germs, enhance the wound-healing environment, hydrate and soften necrotic tissue, and clean the wound, all while releasing silver ions [72]. Bacteria that are negatively charged are drawn to silver ions because of their negative charge, which enhances the permeability of the outer membrane and causes apoptosis. Toxic effects on the fibroblasts of patients with diabetes may limit their cell activity and collagen production and may dramatically alter the cell’s shape due to silver dressing. Silver dressings dramatically reduce odor, alleviate pain-related symptoms, decrease wound exudate, and have a more extended dressing wear period than other treatments in nonhealing and infected chronic wounds [73].

In order to promote the growth of new tissue and to speed the healing process, autolytic debridement is used [74]. Proteolysis is facilitated by the autolytic breakdown of dead or diseased tissue due of endogenous proteolytic enzymes. Hydroxylated starch and alginates, and hydrospheres are only a few of the materials that may be used in clothing. Absorbent dressings are used to treat wet wounds, which are intended to absorb wounds [75]. Even with the two types of dressings, all are equally successful in hastening the process of healing [76].

### 7.3. Types of Antibacterial Agents Used to Treat DFUs

For chronic wounds, topical antimicrobials are not suggested because of their inability to maintain a stable moisture balance and autolytic debridement. Because of their minimal toxicity to the host tissue, topical antimicrobials are not favored. Many topical antiseptics/antimicrobials such as 10 percent solution for povidone iodine, chlorhexidine, acetic acid (5 percent), treatment with compounds containing silver, and hydrogen peroxide (H_2_O_2_) for DFIs are were used [20,76,77].

### 7.4. Systemic Therapy with Antibiotics

When symptoms of localized, progressing, or systematic infections occur, systemic antibiotic treatment is suggested. The course of administration and antimicrobial agent to be employed is determined by the results of a microbiological culture, the clinical symptoms, the body composition, and thet patient’s immune competence [63]. It is common practice to start with a broad-spectrum antibiotic during normal therapy before moving on to one with a more narrow focus once the results of the bacterial culture are clear. Extreme, non-responsive, spreading infections or suspicions of significant osteomyelitis may need hospitalization and intravenous antibiotic (IV) treatment [44]. Gram-positive *staphylococci* and *streptococci* may be treated with oral antibiotics. If a particular antibiotic fails to treat the infection, a second one is injected. If the patient has a history of infection, if the community has a high frequency of MRSA infection, or if the illness is resistant to medicine, empirical MRSA treatment may be considered [78]. IDSA suggests antibiotics for one to two weeks for mild infections and for two to three weeks for moderate to serious infections, although antibiotics will normally be stopped after clinical signs and effects of infections are resolved. The broad-spectrum drugs most often used are beta-lactam or beta-lactamase inhibitor combos, such as piperacillin/tazobactam, ampicillin/sulbactam, and ticarcillin/clavulanic acid [79,80,81].

## 8. Some Emerging Therapies in Brief for the Treatment of DFUs

Several new therapies are being developed to speed up healing of the ulcer and they differ from the usual DFU therapy. Some examples include the use of adjuvant growth factors, inflammatory modulators, herbal extracts, and blood products; biological treatment; hazardous pressure injuries; hyperbaric oxygen therapy; and skin replacements. Supplemental treatments, on the other hand, do not replace the requirement for regular diabetic foot care [82,83,84,85,86,87]. Current treatment plans for DFUs include enhanced adjuvant treatments. To treat these resistant ulcerations, biologic therapies, such as recombinant growth factors, platelet-rich plasma (PRP), and other treatments, may be essential to inducing healing, avoiding limb loss, and enhancing the quality of life for patients. Recombinant platelet-derived growth factor and a pair of cell-based treatments (bioengineered skin equivalents and dermal substitutes) are the biologic therapies for DFUs with the most scientific backing. Larger wounds, more severe wound grades, longer duration, and a longer time to treatment with advanced biologic therapies have all been linked to a longer time to healing, independent of the advanced biologic treatment employed [88,89].

**Figure 4:**
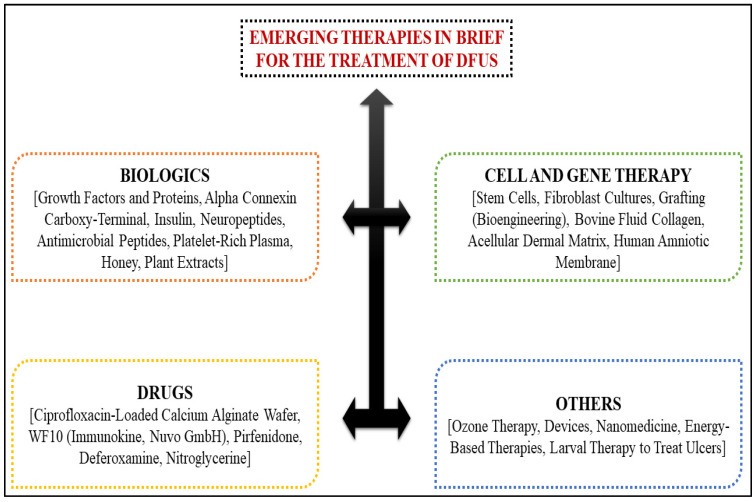
A collection of innovative treatments for DFUs that have been published in the literature.

Using stem cell therapy to treat DFUs has emerged as a possible treatment option. Cell recruitment, immunomodulation, extracellular matrix remodeling, angiogenesis, and neuroregeneration are all promoted by the cytokines produced and secreted by stem cells, all of which aid in wound healing and tissue regeneration. Stem cells can differentiate into various cell types, including keratinocytes, myofibroblasts, pericytes, and endothelial cells. For certain patients who have exhausted all other revascularization options, stem cell therapy is presently employed as an alternative to amputation. The design of the subsequent randomized clinical trials may be aided by the agreement between preclinical and clinical investigations [90,91]. Farideh Davani et al. developed vancomycin and imipenem/cilastatin-loaded core–shell nanofibers to facilitate the treatment of DFUs [92]. Oral antibiotics that cover skin flora such as *streptococci* and *Staphylococcus aureus* may treat patients with minor infections in outpatient settings. Effective options include medications such as cephalexin, dicloxacillin, amoxicillin-clavulanate, or clindamycin [93]. There are a number of innovative treatments for treating DFUs that have been published in the literature (Figure 4). 

## 9. Recent Upgrade in the Field of DFUs

Maggot debridement treatment is the deliberate application of live, “medical-grade” fly larvae to wounds to induce debridement; disinfection; and eventually, wound healing. It is well recognized that maggot treatment may be used in chronic wounds to eliminate necrotic tissue, to promote the growth of granulation tissue, and to eradicate germs. This therapy has been utilized as an alternative to traditional treatments for DFUs plagued by bacterial resistance [94,95]. A 74-year-old female patient with diabetes for more than 30 years was treated with maggot treatment utilizing *Chrysomya megacephala* larvae. Microbiological samples were taken to determine the cause of the illness. The 43-day treatment resulted in a decrease in necrosis and a 0.7 cm^2^ retraction of the ulcer [96]. Using a combination of surgical debridement, maggot treatment, negative pressure wound therapy (NPWT), and silver foam dressings, Naser Parizad et al. presented their experience treating and maintaining patients with DFU. Patient’s ulcers were completely cured after three months and ten days, and they were released in excellent health. They detected multidrug-resistant bacteria in the patient’s lesions, including *Staphylococcus aureus* [97].

NPWT and typical saline dressings have been compared in the treatment of DFU by Haraesh Maranna et al., and 45 patients with DFUs of grades 1 and 2 were included in this randomized controlled experiment. Twenty-two patients in group A received NPWT, while twenty-three patients in group B received saline dressings. NPWT reduced the size of the ulcer, enhanced the development of granulation tissue, shortened the hospital stay, and resulted in a lesion that was completely healed. As a result of the prevalence of DFUs in low- and middle-income countries such as India, early healing allows patients to return to their daily routines [98]. Patients with DM and DFUs had their cutaneous microvascular function tested to see how recurring transcutaneous infusion of gaseous CO_2_ (CO_2_ treatment) affected it. Patients with DM benefit from repeated CO_2_ treatment because it improves microvascular function without causing systemic harm [99]. Using a new method called PTCTD (proximal tibial cortex transverse distraction), doctors have been able to treat DFU with promising results in terms of wound healing and the prevention of amputation. The chemokine stromal cell-derived factor-1 (SDF-1) may have a role in increasing neovascularization in addition to osteogenesis when bone displacement occurs, according to previous research. SDF-1, a chemokine that plays a crucial role in neovascularization and homing, is a key player in the migration of endothelial progenitor cells (EPCs) and mesenchymal stem cells (BMSCs). Experiments conducted in the lab and on animals have shown that bone distraction increases the expression and plasma levels of SDF-1. SDF-1 deficiency has been linked to poor neovascularization in patients with DFUs and wounds, according to certain studies [100].

Autologous platelet-rich plasma (PRP) is becoming more commonly used in the treatment of DFUs [101]. PRP preparation is prohibitively expensive and time-consuming, making it unsuitable for widespread use. L-PRF (leukocyte- and platelet-rich fibrin) is predicted to be used more often in the future since it is simple and inexpensive. It is possible to wait 1–2 weeks between L-PRF injections because the fibrin network in L-PRF serves as both a biological matrix for tissue regeneration and a release site for growth factors that are released over time. L-PRF is a viable treatment option for diabetics with chronic wounds because of its many advantages [102]. Adipose-derived mesenchymal stem cell injection into chronic DFUs was studied for its safety and effectiveness. It has been shown that allogeneic adipose-derived mesenchymal stem cell injections are a safe and effective therapy for chronic DFUs, accelerating wound healing [103]. Clinical trials were conducted on patients with DFUs to determine the therapeutic effect of continuous oxygen diffusion (CDO) combined with conventional moist wound dressing (MWD). The research found that the combination group had a faster rate of wound healing, a lower white blood cell count, and lower levels of high-sensitivity C-reactive protein compared with the MWD and CDO groups. During a one-year follow-up, the combination group had an amputation rate of 0%, which was much lower than the other two groups. The combination of MWD and CDO was beneficial in encouraging healing and reducing infection in DFUs, suggesting that it may represent a novel method for treating this serious clinical problem [104]. In the case of individuals with DFUs who underwent surgical off-loading concurrently with foot ulcer closure and did not experience recurrence for a period of two years following surgery, there were no signs of recurrence of the foot ulcer after two years, and the patients were able to carry out daily tasks on their own after that time. It is less likely to interfere with daily activities if both reconstructive surgery and surgical off-loading are performed at the same time, and it helps to reduce ulcer recurrence [105].

The rising prevalence of extensively drug-resistant bacteria (XDR) in individuals with chronic DFU poses a major hazard of foot amputation. The optimal dosage estimates for currently available drugs are becoming insufficient against widespread drug-resistant pathogens. The use of antibiotic concentration regimes has been overlooked due to the resistance mechanisms of the potent pathogens, and as a result, piperacillin monotherapy, piperacillin-tazobactam, ceftalozane-tazobactam, etc., have been recommended for a long time as a treatment for persistent cases of DFU. Two isolates, VIT PC 7 and VIT PC 9, were found to be resistant to all five classes of antibiotics, indicating widespread XDR. *Pseudomonas aeruginosa* VIT PC 7 and VIT PC 9 whole-genome sequence analysis revealed the existence of many RND efflux and antibiotic resistance genes. The MICs for ciprofloxacin and meropenem were determined using the broth microdilution technique. A checkerboard analysis was used to conduct a synergistic test, and the fractional inhibitory concentration index (FICI) was used to estimate a sub-MIC concentration of ciprofloxacin/meropenem. On the other hand, a test employing antibiotics at or below their minimum inhibitory concentration (MIC) showed the largest reduction in biofilm-forming cells, which proves the effectiveness of both medicines. Thus, by utilizing optimal ciprofloxacin/meropenem concentrations in chronic conditions such as diabetic foot ulcers, an expansion of the antimicrobial spectrum can be accomplished. In addition, sub-MIC doses of ciprofloxacin/meropenem may be a promising alternative for predicting the continuing drug-resistant problem [106].

Vancomycin and imipenem/cilastatin-loaded nanofibers with a core–shell structure were developed to aid in the treatment of DFUs. Due to the unique core–shell nanofibers, electrospinning was utilized to produce nanofibers composed of polyethylene oxide in the shell compartment, chitosan in the core compartment, and imipenem/cilastatin in the shell compartment. Testing various drug-loaded nanofibers against *MRSA, Escherichia coli*, and *Pseudomococcus aureus* using disc diffusion, the nano-fibrous mats showed significant antibacterial activities against *S. aureus* and MRSA, with inhibition zones of 2.9 and 2.5 cm, respectively, and against gram-negative bacteria *E. coli* and *P. aeruginosa*, with inhibition zones of 1.9 and 2.8 cm, respectively. Due to these nano-fibrous mats’ strong antibacterial activities, they may be employed as effective medication delivery systems not only for DFU infections but also for other chronic wounds [107].

Adjuvant hyperbaric oxygen therapy (HBOT) improved ulcer healing and decreased the rate of amputation in individuals with non-healing DFUs. HBOT is a reasonably safe intervention [108]. The modified tibial transverse transport (mTTT) technology was used to treat diabetic ischemic DFU in individuals with T2DM, and the technique’s efficacy and safety were evaluated. The patients did not experience significant amputation, recurrence, or treatment-related problems. mTTT can be used efficiently and safely to treat ischemic DFUs in patients with type 2 diabetic. This technique is a critical component of the ischemia DFU therapy system and needs additional investigation [109]. The fabrication of silver nanoparticles (AgNPs) using an aqueous extract of *Turbinaria conoides* (TC) was investigated by the reduction of Ag+ ions in a silver nitrate solution. The TCAgNPs showed significant antibacterial efficacy against multidrug resistant isolates of DFUs such as *Staphylococcus aureus*, *Klebsiella pneumoniae*, *Pseudomonas aeruginosa*, and *Enterococcus faecalis* using disc diffusion and the least inhibitory concentration method. The development of fresh formulations containing TCAgNPs has been suggested as a possible alternative healing method for diabetic foot infections [110].

Multicenter randomized controlled trials (RCTs) have recently revealed novel evidence-based medicines. Such evidence may be found in the treatments for neuro-ischemic ulcers that use sucrose octasulfate and in the treatment for ulcers with or without ischemia that use a multi-layered patch of autologous leukocytes, platelets, and fibrin. There is also good RCT evidence for placental-derived products, as well as topical and systemic oxygen therapies in the healing of ulcers [111]. The use of micro- and nanoformulations of biomaterials in wound dressings has recently showed increased therapeutic properties [112,113]. Carboxymethyl, dialdehyde, and 2,2,6,6-tetramethylpiperidine-1-oxyl-oxidized celluloses are typical biomaterials with superior physicochemical and medicinal qualities compared with unmodified cellulose [114]. A high prevalence of the pathogen *Staphylococcus aureus* was found in the DFU. Real-time polymerase chain reaction (RT-PCR) is the most reliable method for detecting and confirming MRSA infection, despite the use of cefoxitin and oxacillin disc diffusion [115]. Dasman Diabetes Institute (DDI) clinic patients in Kuwait were analyzed to determine the microbiological profile of DFUs, which are often seen in patients with type 2 diabetes. Gram-positive and Gram-negative bacteria were found in comparable numbers in both sexes, regardless of age or glucose levels. Gram-positive bacteria predominate in ulcers free of ischemia, while Gram-negative bacteria predominate in ulcers affected by ischemia. *Pseudomonas aeruginosa* was more frequent in ulcers with infection and ischemia than *Staphylococcus aureus* was in ulcers without ischemia [116]. The microbiological profile of DFU in Lebanon is similar to that of other nations in the Middle East and North Africa (MENA) area, with significant variances when compared with that of the Western world. Because of this, it is critical to define regional antimicrobial treatment recommendations for use in hospitals. Considering the high incidence of aerobic gram-negative rods (GNR) in DFU, in addition to the high prevalence of fluoroquinolone resistance, the selection of empiric antibiotics should be considered. Except in the case of individuals with certain risk factors, empiric therapy for MRSA or *Pseudomonas* does not seem to be essential [36].

## 10. Concluding Remarks

When people with diabetes develop foot ulcers and infections, they risk their health and their lives in danger. Diabetes neuropathy, vasculopathy, immunopathy, and inadequate glucose control all contribute to the development of diabetic foot disease. A thorough clinical exam of the patient is the first step in making a correct diagnosis of a diabetic foot. This is followed by early care that focuses on prevention. Essential preventive measures include education; regular follow-ups; and direct coordination among a multidisciplinary team of doctors, hospitalists, endocrinologists, infectious disease specialists, and wound treatment experts. Further multicenter randomized controlled trials must inform on treatment decisions and intervention approaches. As a public health issue with a significant influence on a patient’s quality of life, chronic wounds resulting from diabetes are treated with various treatments (biological, technologies, and pharmaceuticals) that have been proven to be successful. Since none of these treatments accomplish the main aim of complete wound healing, the FDA does not approve of them; thus, more controlled studies are required to evaluate their effectiveness. Further research is necessary for the different phases of DFUs to benefit from a combination of cell- and gene-based therapy. Because DFUs are caused by a multitude of distinct pathogenic pathways, a monotherapy approach would result in an exceedingly low recovery rate. Therefore, the treatment of DFU requires a multimodal and interdisciplinary approach.

## Data Availability

Not applicable.

## Abbreviations

IDF	International Diabetes Federation
DFU	Diabetic foot ulcers
PAD	Peripheral arterial disease
ROS	Reactive oxygen species
NADPH	Nicotinamide adenine dinucleotide phosphate
DFIs	Diabetic foot infections
IDSA	Infectious Diseases Society of America
SIR	Systemic inflammatory reaction
CCO	Clostridial collagenase ointment
NPWT	Negative pressure wound therapy
LD	Laser Doppler
LTH	Local thermal hyperemia
PTCTD	Proximal tibial cortex transverse distraction
EPCs	Endothelial progenitor cells
BMSCs	Bone marrow-derived mesenchymal stem cells
PRP	Platelet-rich plasma
CDO	Continuous diffusion of oxygen
MWD	Moist wound dressing
XDR	Extensively drug-resistant bacteria
mTTT	Modified tibial transverse transport
AgNPs	Silver nanoparticles
TC	Turbinaria conoides
RCTs	Multicenter randomized controlled trials
